# Combination of SAXS and Protein Painting Discloses the Three-Dimensional Organization of the Bacterial Cysteine Synthase Complex, a Potential Target for Enhancers of Antibiotic Action

**DOI:** 10.3390/ijms20205219

**Published:** 2019-10-21

**Authors:** Brenda Rosa, Marialaura Marchetti, Gianluca Paredi, Heinz Amenitsch, Nina Franko, Roberto Benoni, Barbara Giabbai, Maria Giovanna De Marino, Andrea Mozzarelli, Luca Ronda, Paola Storici, Barbara Campanini, Stefano Bettati

**Affiliations:** 1 Dipartimento di Medicina e Chirurgia, Università di Parma, Via Gramsci 14, 43126 Parma, Italy; brenda.rosa@studenti.unipr.it (B.R.); roberto.benoni@uochb.cas.cz (R.B.); mariagiovanna.demarino@studenti.unipr.it (M.G.D.M.); luca.ronda@unipr.it (L.R.); stefano.bettati@unipr.it (S.B.); 2 Centro Interdipartimentale Biopharmanet-TEC, Università di Parma, Parco Area delle Scienze 27/A, 43124 Parma, Italy; marialaura.marchetti@unipr.it; 3 Centro Interdipartimentale Siteia, Università di Parma, Parco Area delle Scienze 181/A, 43124 Parma, Italy; gianluca.paredi@unipr.it; 4 Institute for Inorganic Chemistry, Graz University of Technology, Stremayrgasse 9, 8010 Graz, Austria; heinz.amenitsch@elettra.eu; 5 Dipartimento di Scienze degli Alimenti e del Farmaco, Università di Parma, Parco Area delle Scienze 23/A, 43124 Parma, Italy; nina.franko@studenti.unipr.it (N.F.); andrea.mozzarelli@unipr.it (A.M.); 6 Structural Biology Laboratory, Elettra Sincrotrone Trieste S.C.p.A., SS 14 km 163,5 in AREA Science Park—Basovizza, 34149 Trieste, Italy; barbara.giabbai@elettra.eu; 7 Istituto di Biofisica, CNR, Via Moruzzi 1, 56124 Pisa, Italy; 8 Italian National Institute of Biostructures and Biosystems, Via Medaglie d’Oro 305, 00136 Rome, Italy

**Keywords:** cysteine biosynthesis, cysteine synthase complex, SAXS, protein painting, serine acetyltransferase, *O*-acetylserine sulfhydrylase

## Abstract

The formation of multienzymatic complexes allows for the fine tuning of many aspects of enzymatic functions, such as efficiency, localization, stability, and moonlighting. Here, we investigated, in solution, the structure of bacterial cysteine synthase (CS) complex. CS is formed by serine acetyltransferase (CysE) and *O*-acetylserine sulfhydrylase isozyme A (CysK), the enzymes that catalyze the last two steps of cysteine biosynthesis in bacteria. CysK and CysE have been proposed as potential targets for antibiotics, since cysteine and related metabolites are intimately linked to protection of bacterial cells against redox damage and to antibiotic resistance. We applied a combined approach of small-angle X-ray scattering (SAXS) spectroscopy and protein painting to obtain a model for the solution structure of CS. Protein painting allowed the identification of protein–protein interaction hotspots that were then used as constrains to model the CS quaternary assembly inside the SAXS envelope. We demonstrate that the active site entrance of CysK is involved in complex formation, as suggested by site-directed mutagenesis and functional studies. Furthermore, complex formation involves a conformational change in one CysK subunit that is likely transmitted through the dimer interface to the other subunit, with a regulatory effect. Finally, SAXS data indicate that only one active site of CysK is involved in direct interaction with CysE and unambiguously unveil the quaternary arrangement of CS.

## 1. Introduction

Organization of proteins and especially enzymes in multiprotein assemblies responds to many functional and structural requirements, from substrate channeling to regulation, from spatial co-localization to stabilization of poorly stable conformational states [1,2,3]. Proteins organize in complexes of different size and localization, which can be either stable or transient and add further complexity to cell function and regulation. The relevance of protein complexes for understanding cell physiology and pathology is witnessed by the ever-increasing number of studies aimed at mapping the human and bacterial interactomes [4,5,6,7,8,9,10], and by the recent development of drugs targeting protein–protein interactions (PPIs) [11]. No exception to this rule is the field of antibiotic discovery that has witnessed a resurgence due to the relentless emergence of resistance [12,13,14,15,16]. In this scenario, the knowledge of the structure and function of bacterial complexes is indeed considered relevant for directing medicinal chemistry efforts.

**Figure 1:**
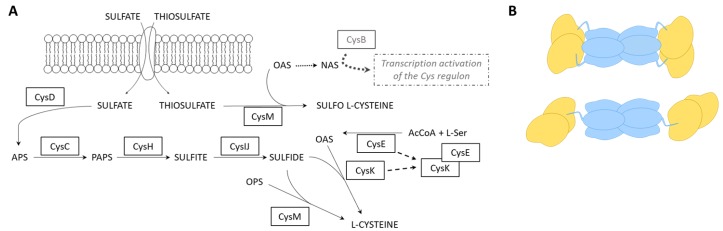
The role of cysteine synthase (CS) in cysteine biosynthesis in *Escherichia coli*. (**A**) The reductive sulfate assimilation pathway is composed of eight enzymes (in black, boxed) that catalyze the synthesis of L-cysteine starting from sulfate/thiosulfate. *N*-acetylserine (NAS) spontaneously forms from *O*-acetylserine (OAS) by an intramolecular *O*-to-*N*-acetyl shift. It binds to the transcriptional activator CysB and induces the expression of the cysteine operon. *O*-phosphoserine (OPS) is an alternative substrate of *O*-acetylserine sulfhydrylase isozyme B (CysM); (**B**) Sketch of the two alternative models that have been proposed for the quaternary arrangement of CS [39], consistent with the observed stoichiometry. In the upper drawing, both active sites of the *O*-acetylserine sulfhydrylase isozyme A (CysK) dimer (yellow) are engaged by the C-terminal peptide of two subunits of the serine acetyltransferase (CysE) trimer (cyan). In the bottom drawing, only one active site of the dimer is involved in complex formation, while the second is unoccupied and available for catalysis.

Cysteine metabolism (i.e., de novo biosynthesis and degradation) [17], is intimately connected with many bacterial functions relevant to infection, such as resistance to oxidative stress and resistance to antibiotics [18,19,20,21,22], biofilm formation [23], and toxin activation [24,25]. For this reason, this metabolic pathway has received much attention in the last ten years as a potential target for the development of antibiotics or antibiotic enhancers [17,26,27,28,29,30,31,32,33,34,35,36,37,38]. Cysteine biosynthesis in bacteria is performed through eight enzymes plus several permeases [17] (Figure 1A) that allow entry of sulfate/thiosulfate inside the cell.

Only four of these enzymes have been the target of significant medicinal chemistry efforts, either in enteric bacteria or in *Mycobacterium tuberculosis*: *O*-acetylserine sulfhydrylase isozymes A and B (CysK/CysM) [35,36,37,40,41], phosphoadenosine phosphosulphate reductase (CysH) [38], and serine acetyltransferase (CysE) [42]. Although medicinal chemistry campaigns have successfully led to the discovery of very potent enzyme inhibitors [17,29], the translation of these inhibitors to molecules with antibacterial activity requires more effort [37,38,40]. One important aspect of the cysteine metabolism is the ability of some enzymes of the pathway, namely CysK, CysE, ATP sulfurylase (CysD) and CysD-associated GTPase (CysN), to form complexes. Particularly relevant to this work is the complex formed by CysK and CysE, the so-called cysteine synthase (CS) [43,44,45,46,47,48,49,50,51,52]. CS is stabilized by the insertion of the unstructured and flexible C-terminal sequence of CysE into the active site of CysK that is thus, inhibited when inside the complex [39,49,53]. Since inhibition of CysK activity by CysE does not support a channeling function for the complex, other hypotheses have been proposed over the years, including CysE stabilization towards cold inactivation and proteolysis, or activation of CysE [54,55,56]. Our group first established that CS formation is a process that involves at least two steps where the rate of complex assembly is limited by a slow conformational change [51,52]. Complex formation leads to an almost full inhibition of CysK that retains about 10% of activity and to the alleviation of substrate inhibition on CysE, which is, however, of uncertain physiological significance [50]. The complex is dissociated by *O*-acetylserine (OAS) and stabilized by bisulfide [52,57,58], a property that suggests a potential role as a sensor of sulfur supply to the cell [59]. Despite an extensive characterization of CS from *Haemophilus influenzae* [39,51,53], *E. coli* [49,50,60,61], *Salmonella enterica* serovar Typhimurium [43,62], and plants [47,48,58,63,64,65,66,67], still many questions are unanswered on its function, regulation, structure, and dynamics. For instance, complex stoichiometry is in principle consistent with two structural models (Figure 1B), one in which two C-terminal peptides of CysE bind the two opposite active sites of CysK dimer, and another in which only one CysK active site is occupied. This latter model is better supported by data that show partial inhibition of CysK activity by CysE, even at saturating CysE concentrations [43,50,68]. In this model CysE competitively inhibits one CysK active site by direct binding to the OAS binding pocket and exerts non-competitive inhibition on the unoccupied CysK active site. This model requires a long-range allosteric communication between the two active sites of the CysK dimer upon CS formation. Additionally, an allosteric communication has been proposed to take place between the two dimers of trimers that compose CysE, that might be modulated by CysK binding [69].

**Figure 2:**
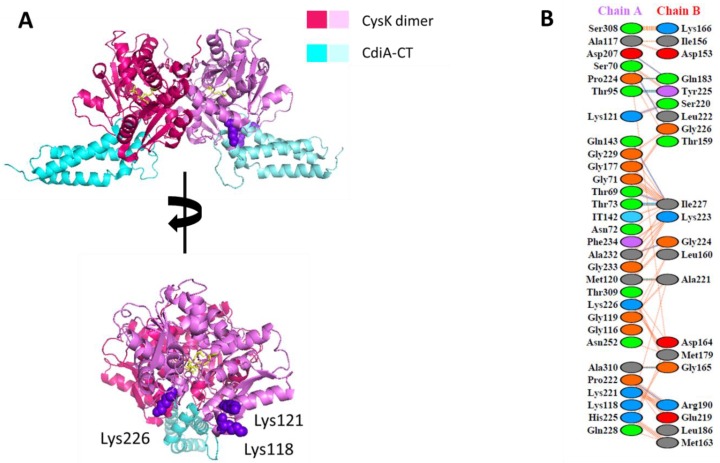
Structure analysis of the complex between CysK and the C-terminal region of Contact-dependent growth inhibition A protein (CdiA-CT) from *E. coli*. (**A**) Three-dimensional structure of CysK/toxin complex (Protein Data Bank code 5j43). Lys118, Lys121, and Lys226 (see (B)) are shown in violet and space-fill mode. Pyridoxal 5′-phosphate (PLP) is shown in yellow. The toxin monomer has been removed from the side view of the structure (lower panel). (**B**) Residue interactions across the interface between CysK and CdiA-CT as calculated by PDBSum (http://www.ebi.ac.uk/pdbsum/) on Protein Data Bank (PDB) entry 5j43. Chain A represents one subunit of CysK dimer, chain B represents one CdiA-CT monomer. H-bonds are depicted in blue, non-bonding contacts are depicted in orange. Four Lys residues are involved in interactions across the interface, namely Lys121, Lys226, Lys221, and Lys118. Lys221 is not a cleavage site recognized by trypsin because it is followed by a Pro residue. Residue color-code: positive, blue; negative, red; neutral, green; aliphatic, grey; aromatic, violet; Pro and Gly, orange.

Recently, Hayes and co-workers discovered that CysK is the permissive factor that activates a bacterial tRNase, the C-terminal region of Contact-dependent growth inhibition A protein (CdiA-CT) toxin in an uropathogenic strain of *E. coli* [25]. The authors, in addition to the remarkable finding of a moonlighting function for CysK, proposed that the toxin forms a complex with CysK using the same structural motif used by CysE (i.e., insertion of the C-terminus in CysK active site). Further structural [70] and functional [24] studies confirmed the original finding and shed light on the details of complex formation and toxin activation. CysK/CdiA-CT complex is formed by two toxin monomers that bind to one CysK dimer with the C-terminal carboxylate engaging to the same binding site that is occupied by the substrate of the enzyme (Figure 2A).

CysK/toxin and CS complexes have roughly the same K_d_ of about 5 nM and are apparently not in competition with each other [24]. Despite many attempts, the three-dimensional structure of CS has not been solved yet by crystallographic methods, and CysK/CdiA-CT complex represents the first multiprotein complex involving solved CysK to date. For this reason, CysK/CdiA-CT complex represents a fundamental model and an obligate starting point towards the goal of solving CS structure.

We believe that understanding the structure and the regulatory role of CS is of particular relevance in the development of potential antibiotics, in light of the observation that inhibition of one enzyme does not only affect the activity of the main target but has also “pleiotropic” effects due to complex perturbation, most of which are, at the moment, unpredictable. For example, high-affinity inhibitors of CysK might potentially lead to complex dissociation. Indeed, it has been reported that CysE mutants, deficient in CS formation, induce cysteine auxotrophy in *Salmonella* [71]. Furthermore, since all CysE activity is within the complex [57,72], its disruption would free the protein in the cellular milieu where it could find a different binding partner or could be degraded. Since targeting complex formation, rather than the single enzymes, could eventually be revealed as a productive approach, knowledge of the hotspots for complex formation is of high significance. So far, only OAS has been identified as a small molecule able to dissociate CS, both from bacteria and plants [43,73]. To our knowledge, no other report describes small molecules capable of interfering with complex formation, with either a stabilizing or destabilizing effect. This work has the aim to gain structural information on the CS in solution and to connect this information with functional data to answer three questions: i) What regions of CysK are involved in the protein–protein interaction? ii) What is the geometry of CS, and which of the two possible binding modes of CysK is compatible with it? iii) Does complex formation induce any long-range conformational changes in the protein that could account for an allosteric control of enzyme activity?

## 2. Results and Discussion

### 2.1. Validation of the Protein Painting Assay

**Scheme 1:**
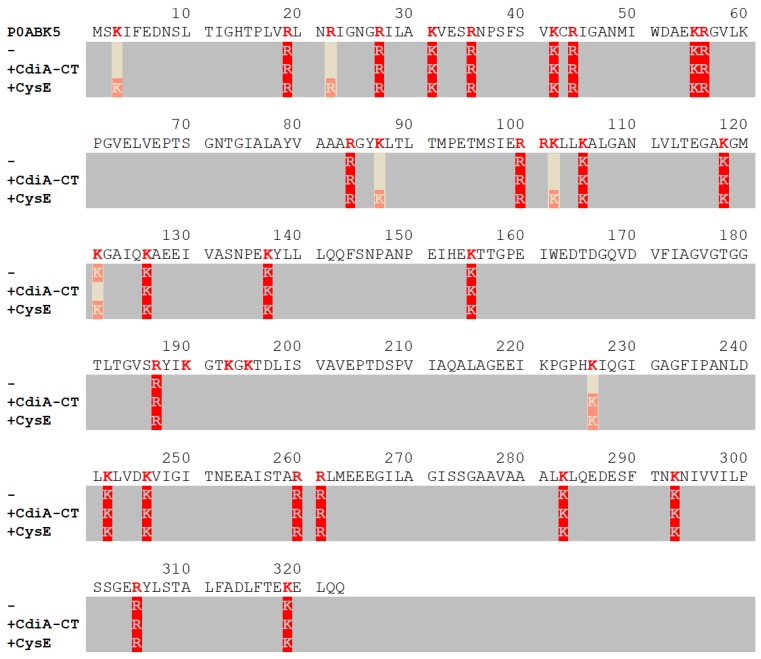
Trypsin cleavage sites on CysK. The sites recognized and cleaved by trypsin on the unpainted CysK are shown in the upper sequence (UniProtKB P0ABK5) in bold red. The sites that are recognized on the painted protein, either alone (–) or in complex with toxin (+CdiA-CT) and CysE (+CysE) are indicated with a red background. Trypsin cleavage sites that are differentially recognized when CysK is alone or in the complex are marked with yellow shading. Sites that are cut only within the complex are those that become buried upon complex formation (K3, R22, K87, K102, and K226).

Protein painting is a recently developed technique [74,75] that consists of treating a protein or a protein complex under native conditions with molecular dyes to mask cleavage sites of trypsin, an endopeptidase generally cutting at the C-terminal side of arginine and lysine residues. The dyes stay bound to the protein during the typical denaturation/reduction/alkylation procedure that precedes treatment with trypsin. As a result, sites that are covered by dyes are not cleaved by trypsin and the corresponding peptides are not identified by mass spectrometry. Conversely, sites that are inaccessible to dyes on the native protein (i.e., protein–protein interaction interfaces and buried areas) are then identified by mass spectrometry analysis of the tryptic peptides (solvent exclusion principle [74]). The ability of protein painting to correctly identify the hot spots for complex formation between CysK and its binding partners was first tested on the CysK/CdiA-CT complex for which a three-dimensional structure is available (pdb code: 5j43, Scheme 1). Digestion of denatured CysK with trypsin in the absence of dyes followed by MS analysis of the tryptic peptides gave a 100% sequence coverage, which indicates that all the 34 potential cleavage sites are recognized and digested by trypsin (Scheme 1). First, we tried a combination of three dyes, namely RBB, AO50, and CR (see Materials and Methods for details and full chemical names) that were previously optimized on carbonic anhydrase [74]. Cleavage sites identified on either CysK alone or in the complex with the CdiA-CT toxin are reported in Scheme 1 on a red background. These sites are those not covered by dyes on the native protein or protein–protein complex.

Analysis of the CysK and toxin complex with PDBSum (http://www.ebi.ac.uk/pdbsum/ [76]) allowed the identification of residues that are hot spots for protein–protein interaction (Figure 2B). A total of 30 residues on CysK and 21 residues on the toxin participate in the formation of the interaction surface, which extends for 1280 Å^2^. On the CysK sequence, four lysine residues (K118, K121, K221, and K226) appear to participate in the interface with the toxin and should, in principle, be inaccessible to dyes (Figure 2).

**Figure 3:**
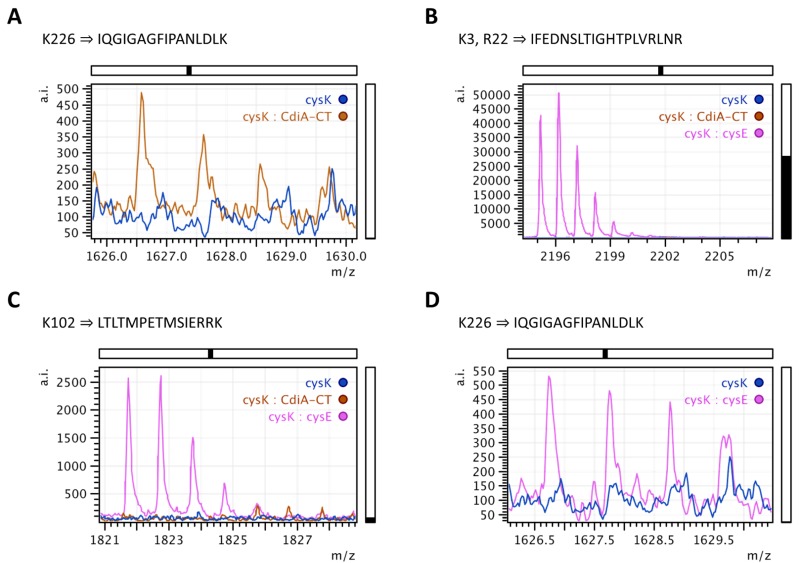
Mass spectra of peptides differentially identified in CysK alone or in the complex with either toxin or CysE. (**A**) Peptide 227–242 from CysK in the complex with toxin and with a theoretical [M+H]^+^ of 1626.61 m/z; (**B**) Peptide 4–22 from CysK in the CS complex with an observed [M+H]^+^ of 2195.22 m/z; (**C**) Peptide 88–102 from CysK in the CS complex with an observed [M+H]^+^ of 1821.78 m/z; (**D**) Peptide 227–242 from CysK in the CS complex with a theoretical [M+H]^+^ of 1626.75 m/z.

K221 cannot be recognized by trypsin because of the presence of a proline residue at its C-terminus; conversely, K118, K121, and K226 are three out of 34 potential trypsin cleavage sites. K226, which is protected by dye labelling from trypsin digestion in CysK (i.e., is solvent-accessible), becomes buried at the toxin interface upon complex formation. The corresponding peptide is indeed identified in the complex (Figure 3A).

K118 and K121 are not covered by the dyes on the uncomplexed CysK (Scheme 1), but, while the K118 cleavage site is identified also on the CysK/CdiA-CT complex, K121 becomes solvent accessible after complex formation. It can be noticed that this residue is in a region of CysK that undergoes extensive conformational changes when the protein binds the substrate (vide infra). It is; thus, possible that K121 becomes solvent-exposed because of a conformational change upon complex formation. When we tried to enhance the surface coverage of exposed trypsin sites by using a different set of dyes, namely 8-Anilino-1-naphthalenesulfonic acid ammonium salt (ANSA), Thioflavine T, Acid Fuchsin, Eosin B (see Materials and Methods), the coverage did not significantly increase. Therefore, we decided not to further pursue the goal to increase coverage, which, in principle, could allow for the identification of other PPI hot spots. Our results are in line with the three-dimensional structure of the complex, where only a small part of the CysK exposed surface (9.2% [77]) is involved in complex formation.

### 2.2. Mapping Protein–Protein Interaction in Cysteine Synthase

**Figure 4:**
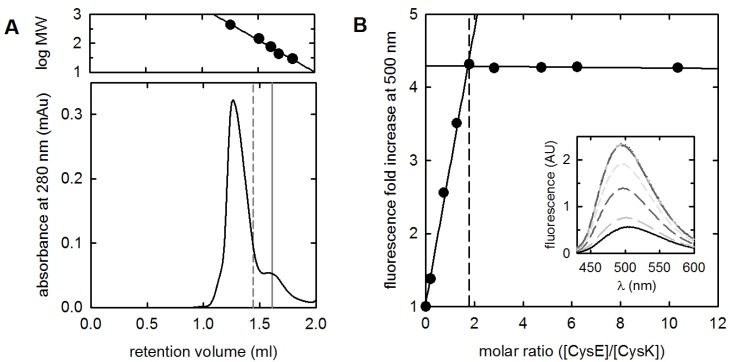
(**A**) Size exclusion chromatography. The chromatogram in black is for 39 µM CysE and in grey for a mixture of 39 µM CysE and 26 µM CysK (1.5 molar ratio). CysE elutes at 1.44 mL (grey, dotted line, corresponding to a molecular mass of 181 kDa) and CysK elutes at 1.61 mL (grey, solid line, corresponding to a mass of 77 kDa). (**B**). Calculation of the binding stoichiometry for the CS by fluorescence emission spectroscopy. The stoichiometric ratio is 1.7. Inset: Fluorescence emission spectra upon excitation at 412 nm in the presence of increasing concentrations of CysE.

Previous works by Hayes’s group and ours demonstrated that the CysK/CdiA-CT complex shares many mechanistic features with CS, among which the occupation of the CysK active site [24,25]. Thus, we applied the protocol used to identify residues involved in protein–protein interaction for the CysK/CdiA-CT complex to CS, for which no three-dimensional structure is available. Complex formation was firstly checked by size-exclusion chromatography and fluorescence spectroscopy (Figure 4).

**Figure 5:**
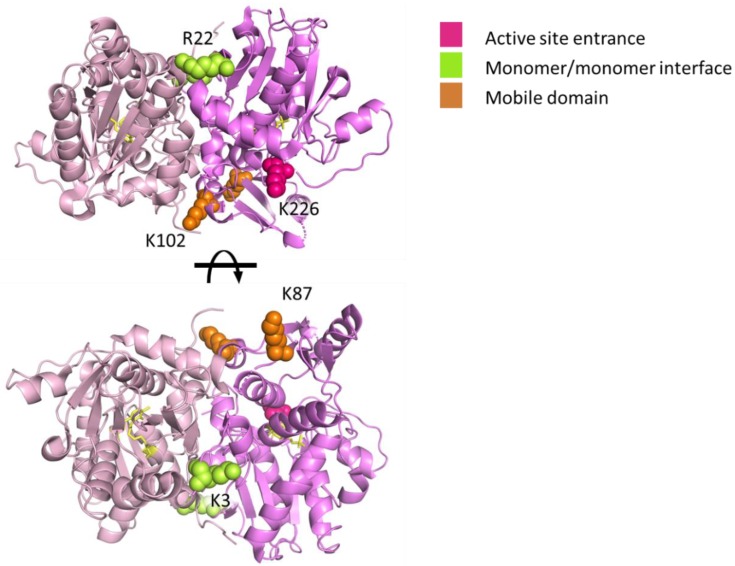
Identification of hot spots for CS complex on CysK three-dimensional structure. The position of K3, R22, K87, K102, and K226 is displayed on the structure of CysK (taken from PDB 5j43). The residues are color-coded according to their position on the three-dimensional structure of CysK. The two CysK dimers are represented in pink shades. The PLP in the active site is shown in yellow in stick mode.

Binding of CysE to CysK can be followed by fluorescence emission spectroscopy since CysK active site occupation by CysE modifies the fluorescence emission properties of the pyridoxal 5′-phosphate (PLP) cofactor [24,39]. The stoichiometric ratio, as calculated by the dependence of PLP emission on CysE concentration, is reached at 1.7, i.e., a CysE hexamer binds two CysK dimers (Figure 4A). A complex formed by mixing a 1.5 molar excess of CysE (predicted molecular mass: 174 kDa) over CysK (predicted molecular mass: 71 kDa) elutes mainly at a calculated molecular weight of 470 kDa, larger than the theoretical MW of 314 kDa. This was also observed in previous works [50] and can be attributed to the elongated shape of the complex. We applied to the CS complex the protein painting method previously tested and optimized on CysK/CdiA-CT. Scheme 1 reports the cleavage sites identified on CysK when it is complexed with CysE. The results on CS allowed the identification of five trypsin cleavage sites that are differently covered when CysK is free or bound to CysE (Figure 3B–D, Figure 5, and Scheme 1).

The identification as a PPI hot spot of K226, that was also identified at the CysK/toxin interface, further supports the highly similar binding mode of the two CysK binding partners. Interestingly, a mutagenesis study on *Arabidopsis thaliana* CysK identified residues K217, H221, and K222 (K221, H225, and K226 in the *E. coli* enzyme) as essential for CS formation [78]. In addition to K226, also K87 and K102 were identified as sites that become buried upon complex formation. Since they are both relatively distant from the active site entrance (Figure 5), it is difficult to rationalize a possible involvement of these residues in direct interactions with CysE. However, it should be noted that at least three different conformations of the homologous CysK from *S. Typhimurium* have been isolated in the crystal: an unligated, open form [79]; a substrate-bound, closed form [80]; and an inhibited, intermediate form [81]. In a previous work and based on functional data, we speculated that the toxin preferentially binds to/selectively stabilizes the open conformation of CysK, whereas CysE binds to the closed conformation [24]. The transition from the open to the closed conformation involves the rotation of the N-terminal domain that closes on the active site [80]. This movement brings into proximity residues that are far apart in the open conformation. This region encompasses the sequence 87-131, that contains K87 and K102, and that becomes buried upon ligand binding. The present results suggest that binding to CysE could induce similar conformational changes on CysK and further support the original proposal that CysE preferentially stabilize the closed conformation of CysK. K3 and R22 are also masked upon complex formation and are located near the interdimer interface of CysK. The fact that these two residues are identified as trypsin cleavage sites only upon complex formation suggests that the dimer interface might be stabilized within CS. This latter finding is particularly interesting since it indicates the existence of an allosteric communication between the two CysK monomers, where the occupation of one CysK active site is communicated to the second subunit through the strengthening of dimer interface. This is in very good agreement with small-angle X-ray scattering (SAXS) data (vide infra) and with previous functional data that suggest a partial closure of the CsyK active site that is not occupied by the C-terminus of CysE in CS.

### 2.3. SAXS Analysis of CysK, CysE, and CS

**Figure 6:**
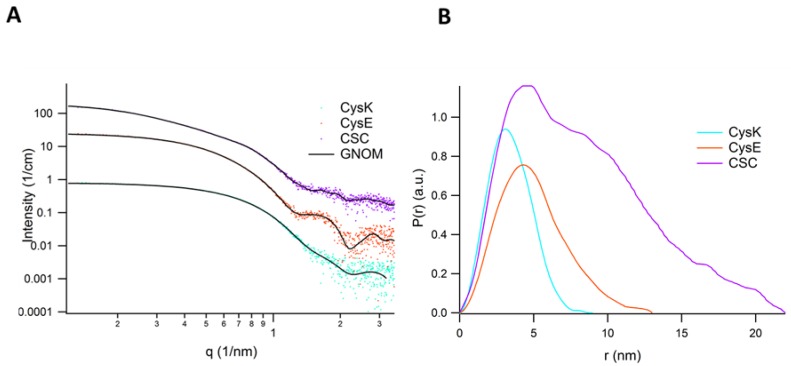
Small-angle X-ray scattering (SAXS) results for CysK (cyan), CysE (orange), and CS complex (CSC, purple)–data shifted vertically for clarity. (**A**) Merged solutions scattering data of CysK, CysE, and CSC (dots), rescaled to 1 mg/mL and shifted vertically for improved representation with the GNOM software fit (black lines). (**B**) Corresponding pair distance distribution function (PDDF) data of GNOM.

SAXS data were collected to further characterize the quaternary structure of the CS complex in solution and compare it with the protein painting results. In order to validate all structures involved in the CS complex, CysK and CysE alone were characterized with SAXS. An overview of the solution scattering data and the corresponding PDDF is presented in Figure 6. The derived structural data (the Guinier radius R_g_, the maximum dimension D_max_, forward scattering I(0), etc.) are reported in Table 1 in comparison with the data obtained for the crystallographic models using CRYSOL [82]. Additionally, the Guinier fits in the corresponding data range are represented in Figure S1, demonstrating the quality of the monodispersed protein samples, while the dimensionless Kratky plot for the same data (Figure S2) assesses the compactness and well-folded protein structures in the region qR_g_ < 10.

**Figure 7:**
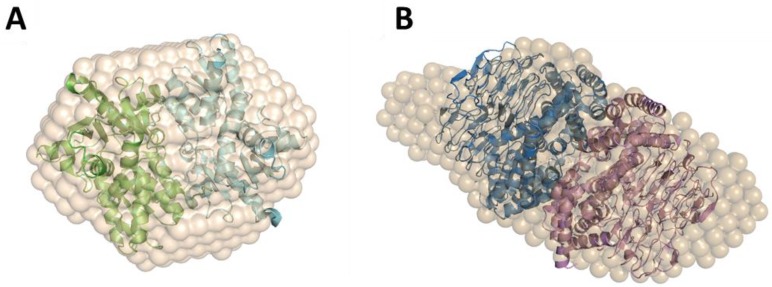
DAMMIF modelling results for CysK and CysE. (**A**) Ab initio DAMMIF-filtered model of CysK (wheat spheres) overlaid with the crystallographic structure (PDB entry 1oas, chain A green and chain B cyan ribbons); (**B**) Ab initio DAMMIF-filtered model of CysE (wheat spheres) with its overlaid hexamer crystal structure using PDB entry 1t3d (trimers shown as blue and magenta ribbons).

Interestingly, both CysK and CysE give solution scattering profiles that are consistent with the known crystal structures [79,84]. The CysK ab initio DAMMIF model, calculated by averaging and filtering, well overlays the dimeric crystal structure and accommodates both the open form (1oas) (Figure 7A) or the closed form (1d6s). For CysE, the bead model nicely fits the shape of the hexameric form, as determined from the 1t3d crystal model (Figure 7B). Intriguingly, at both ends of the SAXS bead model an unoccupied space is visible that could accommodate the C-terminal flexible peptides that were not modeled in the crystallographic structure, but that were present in the protein construct used for SAXS measurements.

**Figure 8:**
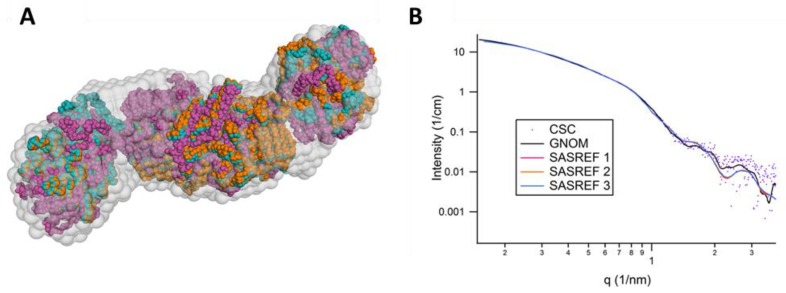
Ab initio model of the CS complex overlaid with the three independent rigid body-refined models resulting from analysis with SASREF program and imposing a constrained distance of 1.5 nm. (**A**) Real-space models of the three independent solutions (each model shown as backbone trace in magenta, orange, and cyan spheres) overlaid with the ab initio model (grey surface, 60% transparent); and (**B**) fitting of the corresponding SAXS pattern for each of the solutions.

To measure reliable SAXS profiles of the CS assembly in solution, the oligomeric protein sample was gel filtered and dialyzed immediately before collecting experimental scattering data. After data reduction and rescaling to 1 mg/mL, the P(r) analysis gave structural parameters of R_g_ = 6.606 ± 0.003 nm and D_max_ = 22.0 nm, and showed an elongated S-shaped envelope (as obtained by DAMMIF) that was compatible with the proposed model sketched in the bottom panel of Figure 1B, in which only one active site of the CysK dimer is occupied by one C-terminal peptide of CysE at each side of the hexamer. The model shown in the top panel of Figure 1B, where both CysK active sites are occupied, was discarded based on the following experimental evidences: i) we performed a simulation of a CS model compatible with the double-occupied CysK dimer (top model in Figure 1B) that resulted in a smaller R_g_ and a D_max_ of 18.5 nm, which is not supported by the experimental data shown in Scheme 1 and in Figure 6B; ii) the number and position of residues identified by protein painting on CysK does not agree with the high surface area of CysK involved in the interaction with CysE in this model; iii) we and other authors [49,50] consistently measured a 10% residual activity of CysK in the presence of a molar excess of CysE, which is not supported by a fully-competitive inhibition model. We believe that one active site of CysK is occupied by the C-terminus of CysE and the other one is allosterically inhibited, yet accessible to the substrate and thus partially active. The molecular weight estimated from the SAXS data is 300 kDa (Table 1), in good agreement with the theoretical one, and within the accuracy expected by SAXS mass determination of proteins in solution [85]. A preliminary CS model was manually constructed using SASpy, starting from PDB models (1oas and 1t3d), and overlaid to the ab initio bead model (see Figure S3). Three independent runs of rigid body refinement were performed with SASREF in the q-range between 0.15 and 3.9 nm^-1^. The information obtained by protein painting was used to define the residues involved in CysK–CysE interaction: K226 on CysK and R242 on CysE. This is part of the conserved PARIV sequence in the last strand of the α-helix that is solvent exposed and thus available for the interaction with CysK. Three different constrained distances, namely 1.0, 1.5, and 2.0 nm, were set between the CA atoms of residues K226 (CysK) and R242 (CysE). Fit was not satisfactory with 1.0 nm (chi^2^ value around 1.99) and 2.0 nm distance (constrain too loose leading to chi^2^ values of 0.85, 1.81, and 6.84), but 1.5 nm resulted in a good fit for each of the three independent solutions obtained (chi^2^ values: 1.22, 1.23, 1.24). The resulting models fitted well into the ab initio real-space envelope, giving a reasonable overlap with the SAXS pattern and the q-space data regularized with GNOM (Figure 8A,B)

**Figure 9:**
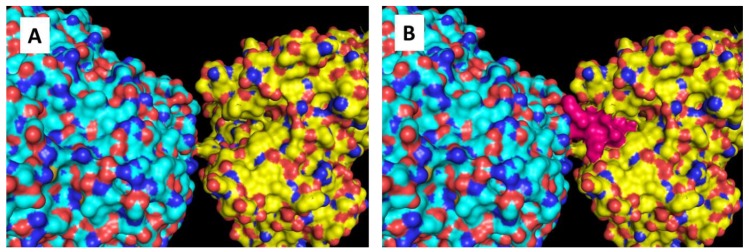
Close-up of the CS complex at the interaction interface between the active site entrance of CysK and the C-terminal end of CysE. CysK (yellow) in complex with CysE (cyan), as obtained from the SASREF rigid body modelling shown in Figure 8 (same orientation), was overlaid with the three-dimensional structure of CysK in complex with the toxin (PDB 5j43) to define the position of the last 11 residues of toxin (KIESALKGYGI). (**A**) A large cavity between CysK and CysE is visible at the entrance of CysK active site. (**B**) When the toxin C-terminal peptide is shown (pink), the cavity appears almost completely occupied by the toxin.

The S-shape of the complex also indicates that the three potential binding sites available to CysK on each protomer of CysE are not equivalent, but that, once CysK binds to one protomer, its binding site on the other protomer is defined. This observation suggests the existence of an allosteric communication between the two trimers within the hexamer. In the model, the CysK active site points towards the C-terminus of CysE but is still solvent accessible (Figure 9A), likely due to the 11 amino-acid C-terminal sequence (INHTFEYGDGI) missing in the deposited crystal structure of CysE. The position potentially occupied by the C-terminal peptide of CysE, which is not visible in any of the three-dimensional structures of the protein solved to date, can be inferred by comparison with the structure of the CysK/CdiA-CT complex. When the structure of CysK in the modeled complex was superimposed with the structure of CysK in the complex with toxin, the last 11 residues of the toxin nicely occupy the large cavity visible in the CS complex (Figure 9B). This finding further supports the consistency of the complex modeled from SAXS data.

## 3. Materials and Methods

Unless otherwise specified, reagents were purchased from Sigma Aldrich (St. Louis, MO, USA) and used as received.

### 3.1. Proteins Expression and Purification

CysK and CysE from *E. coli*, cloned in pET21P and pSH21p vectors, respectively, were over-expressed in the bacterial strains BL21(DE3) and BL21(DE3) Tuner^TM^. Cells were grown at 37 °C in Luria Bertani medium and induced in the presence of 1 mM isopropyl β-d-1-thiogalactopyranoside (IPTG). CysK and CysE were then purified and the tag was removed from CysE as previously described [50], with minor modifications. CysK concentration was determined based on the absorbance of the coenzyme pyridoxal 5′-phosphate (PLP) using an extinction coefficient at 412 nm of 9370 M^−1^·cm^−1^, calculated by the alkali denaturation method [86]. CysE concentration was calculated using an extinction coefficient at 280 nm of 26,900 M^−1^·cm^−1^. The purity of the enzymes assessed by SDS-PAGE was estimated higher than 95%. The specific activity of CysK, determined with the discontinuous assay of Gaitonde [87] in the presence of 0.6 mM NaHS, 10 mM OAS, and 3 nM enzyme (monomer), was 280 U/mg. The specific activity of CysE, determined at 20 °C by measuring the disappearance of acetyl coenzyme A (AcCoA) signal at 232 nm in the presence of 1 mM L-Ser, 0.25 mM AcCoA, and 7 nM enzyme (monomer), was 83 U/mg, in agreement with previously-reported kinetic data [50].

CdiA-CT was expressed in *E. coli* BL21(DE3) Tuner^TM^ and was purified as CdiA-CT:CdiI-His6 complex, as previously described in [88]. CdiA-CT and CdiI-His6 were separated by metal-affinity chromatography in 8 M urea. CdiA-CT was refolded by dialysis into 20 mM sodium phosphate (pH 7.0), 85 mM sodium chloride, 10 mM 2-mercaptoethanol (2-MCE), 2 mM EDTA, and its native structure was evaluated by circular dichroism spectroscopy. The protein was further purified using size-exclusion chromatography (SEC) on a fast protein liquid chromatography (FPLC) column packed with Ultrogel AcA44 resin (exclusion limit 200 kDa, operating range 17–175 kDa, column volume 63 mL and void volume 20.4 mL, Pall Corporation, Port Washington, NY, USA), run at 0.2 mL/min in 20 mM sodium phosphate, pH 7.0, 85 mM sodium chloride, 10 mM 2-MCE, 2 mM EDTA. CdiA-CT concentration was estimated using an extinction coefficient at 280 nm of 13,300 M^−1^·cm^−1^.

### 3.2. Spectroscopy

Absorption measurements were carried out at 20.0 ± 0.5 °C using a Varian (Palo Alto, CA, USA) CARY400 spectrophotometer. All spectra were corrected for buffer contributions. Fluorescence emission spectra were collected using a FluoroMax-3 fluorometer (HORIBA Jobin Yvon, Kyoto, Japan) at 20 ± 0.5 °C, equilibrating samples for 5 min prior to spectra acquisition. CysE/CdiA-CT stoichiometric binding to CysK was monitored by measuring PLP fluorescence emission at 500 nm following excitation at 412 nm (see [24]) [39,50,51]. All spectra were corrected for buffer contribution, and the slit width set to optimize the signal-to-noise ratio.

### 3.3. Size Exclusion Chromatography

The oligomeric state of CysE and CysE/CysK complex in native conditions was determined on an analytical HPLC-SEC Superdex 200 increase 3.2/300 column (GE-Healthcare, Chicago, IL, US) in PBS in the presence of 1 mM tris(2-carboxyethyl)phosphine (TCEP). A calibration curve was obtained by running five commercial standards for size exclusion chromatography (blue dextran, ferritin 440 kDa, conalbumin 75 kDa, ovalbumin 43 kDa, and carbonic anidrase 29 kDa, GE Healthcare) and the home-made standard glyceraldehyde 3-phosphate dehydrogenase (GAPDH, 144.2 kDa).

### 3.4. Protein Painting with Small Molecule Dyes

All protein preparations, either alone or in complex with binding partners, were incubated for 15 min in a solution containing 5 mM of each of the following molecular paints, in accordance with a published protocol [89]: disodium 1-amino-9,10-dioxo-4-[3-(2-sulfonatooxyethylsulfonyl)anilino]anthracene-2-sulfonate (RBB), disodium 4-amino-3-[[4-[4-[(1-amino-4-sulfonatonaphthalen-2-yl)diazenyl]phenyl]phenyl]diazenyl]naphthalene-1-sulfonate (CR), sodium 4-(4-(benzyl-et-amino)-ph-azo)-2,5-di-cl-benzenesulfonate (AO50). Additionally, another set of small-molecule dyes was tested, that included: 8-Anilino-1-naphthalenesulfonic acid ammonium salt (ANSA, K&K Laboratories, New York, MA, USA), 4-(3,6-dimethyl-1,3-benzothiazol-3-ium-2-yl)-N,N-dimethylaniline chloride (Thioflavine T), 2- amino -5- [(4-amino-3-sulfophenyl)(4-imino-3-sulfo-2,5-cyclohexadien-1-ylidene)methyl] -3- methyl-benzenesulfonic acid, sodium salt (Acid Fuchsin), 4′,5′-dibromo-3′,6′-dihydroxy-2′,7′-dinitro-spiro[isobenzofuran-1(3H),9′ [9H]xanthen]-3-one (Eosin B, Merck, Darmstadt, Germany). Dyes were dissolved in PBS and proteins were incubated at room temperature for 15 min. In the case of CysK/CdiA-CT and CS complexes, a pre-incubation of 30 min on ice was added before the incubation with the dyes mixture to allow proper formation of the complex. Proteins were incubated in a total volume of 50 μL at the following concentrations: 5.13 μM CysK alone, 3.33 μM CysK, and 5 μM CdiA-CT for CysK/toxin complex, and 5 μM CysK and 7.5 μM CysE for CS. The relative protein concentrations were chosen based on binding stoichiometries and samples were prepared in triplicate. Excess of molecular paints was removed by acetone precipitation. Briefly, a four-fold sample volume of cold acetone was added to the sample, mixed and incubated for 1 h at −80 °C. Then, the samples were centrifuged for 15 min at 16,000×g at 4 °C and the supernatant discarded. The precipitates were resuspended in 50 μL of PBS. Samples were denatured in 0.5 M urea, reduction was performed in 13 mM DTT for 15 min at room temperature and samples were alkylated with 16 mM iodoacetamide for 15 min at room temperature in the dark. Finally, digestions were performed with trypsin for 2 h at 37 °C. Protein:protease ratio was set to 1:4.4 *w/w* for CysK alone and CysK in CS and 1:3.1 for CysK in complex with CdiA-CT. The reactions were stopped with trifluoroacetic acid (TFA) at 0.1% final concentration and peptides mixtures were desalted using 10 μL Pierce^®^ C18 Tips (Thermo Scientific, Waltham, MA, USA).

Mass spectrometry analyses were carried out using a 4800 Plus MALDI TOF/TOF™ spectrometer (Ab Sciex, Framingham, MA, USA) in positive ion reflectron mode combining 400 shots in the mass range 500–3600 Da. MALDI spots were prepared using the dried droplet method. Briefly, 1 µL of sample was mixed with 1 µL of 10 mg/mL α-cyano-4-hydroxycinnamic acid (HCCA) in 75% *v/v* acetonitrile and 2.5% *v/v* TFA, and 0.5 µL of the solution was spotted onto the plate. Each sample was analyzed in triplicate. External calibration was performed using the following mixture of standard peptides: Bradykinin fragment 1–7 (m/z 757.3997), angiotensin II (human) (m/z 1046.5423), P14R (m/z 1533.8582), ACTH fragment 18–39 (human) (m/z 2465.1989), and insulin chain B oxidized (m/z 3494.6513). Calibrations were accepted at the following conditions: 15 ppm mass tolerance and max outlier error, 4 minimum peaks to match, 5 as minimal signal-to-noise ratio (S/N). Resulting MS spectra were submitted to a homemade database search using the Mascot search engine. Methionine oxidation was selected as variable modification and carbamidomethylation of cysteine as fixed modification, two missed cleavages were tolerated, and peptide mass tolerance was set at 100 ppm. To ensure the quality of database searching results, the peak assignment for every peptide was manually checked, considering S/N = 3 as the limit of detection (LOD).

### 3.5. SAXS Measurements

CysK, CysE, and CS prepared in a stoichiometric ratio were re-purified and buffer exchanged in 20 mM sodium phosphate, 85 mM NaCl, 2 mM EDTA, 10 mM 2-MCE, pH 7.5 using a Superdex 200 10/300 GL (GE-Healthcare, Chicago, IL, US). After the chromatographic run, proteins were dialyzed against their storage buffer, which was later used to record the SAX-scattering baseline. Proteins were concentrated by ultrafiltration and their final concentration was determined by Bradford assay. SAXS measurements were performed using freshly-diluted protein samples at concentrations of 1.5, 1.0, and 0.5 mg/mL. Data sets of samples that showed indication of aggregation were merged with the corresponding data collected at lower concentration.

Small-angle X-ray scattering: Synchrotron SAXS data of all samples were collected on the Austrian beamline at the Elettra Synchrotron (Trieste, Italy) using a Pilatus3 1M detector system at a sample-detector distance of 1.232 m and at a wavelength λ = 0.154 nm. Measurements were carried out at 10 °C in capillaries of 1.5 mm outer diameter/0.01 mm wall thickness made from borosilicate (Hilgenberg, Maisfeld, Germany), enclosed within a custom-made thermostatic compartment connected to an external circulation bath and a thermal probe for temperature control. Raw data were radially averaged and calibrated to absolute units (cm^−1^) by using a freshly-prepared 5 mg/mL BSA solution in 50 mM Hepes pH 7.5. The scattering curves were normalized to the primary beam intensity, corrected for sample transmission, and normalized to absolute scattering units using IGOR Pro (Wavemetrics, Lake Oswego, OR, USA). Each set of scattering patterns was carefully checked and the average after a positive control over radiation damage was performed. Radiation damage was not observed on samples presented in this study. GIFT [90] was used to test for residual constant background. The pair distance distribution function (PDDF) was calculated with GNOM [91], which was also used to determine the radius of gyration and maximum dimension of all protein structures. The data was further validated by Guinier analysis (AUTORG program), and DATPOROD (ATSAS Package) was used to determine the Porod volume and the derived molecular mass (V_P_/M = 1.5). Bead ab initio modeling was conducted using DAMMIF [92], from the ATSAS package. For each run 10 ab initio models were generated and subsequently analyzed and averaged using DAMCLUST and DAMAVER [93] from ATSAS package.

The CysK and CysE crystallographic model structures were superimposed to the ab initio bead models using SUPCOMB [94]. The multi-subunit CS complex was manually fitted in the de novo bead envelope using one hexameric CysE model (1t3d) and two CysK dimers (1d6s) in SASpy [95]. The manual pre-alignment was the starting point for the automatic rigid body refinement with SASREF [96], where a number of putative contact points were imposed as specified in the Results and Discussion, based on experimental data of the model painting.

## 4. Conclusions

Cysteine plays a central role in bacterial metabolism, in the resistance to oxidative stress, in antibiotic resistance, and in biofilm formation. Therefore, cysteine biosynthesis needs a fine-tuning of the involved enzyme activities. Despite the considerable efforts made towards the elucidation of these processes, the subtle mechanisms governing cysteine homeostasis are still poorly understood. Among these, the regulation of CS assembly, its conformation, and its role in CysE activity modulation are still debated. Cysteine biosynthesis is a putative target for enhancers of antibiotic therapy since cysteine-depleted bacteria exhibit a decreased fitness [17]. In particular, CysE, the enzyme involved in the induction of cysteine operon, is a potential novel target. In this work, we exploited protein painting to detect CysK/CysE interaction hotspots in CS and used the information to guide molecular modeling of the two protein structures in the SAXS envelope. Two CysK dimers bind to one hexamer of CysE, with one CysK active site directly involved in binding for each dimer. The occupation of one active site is transmitted through the CysK dimer to the other active site that closes and leads to a 90% inhibition of enzyme activity. Interestingly, the S-shape of the complex suggests that the two interaction surfaces of CysE for CysK are not independent: binding of a CysK dimer to one CysE trimer is allosterically communicated to the second trimer of the CysE hexamer, so that only one possible orientation is allowed for binding of a second CysK dimer. This is in line with an original observation by Hindson and collaborators [69] that the structure of acyltransferases has evolved from trimer to dimer of trimers for regulatory reasons. The elucidation of the CS quaternary structure will pave the way for the discovery of molecules able to interfere with complex formation, which might be useful both as tools to elucidate the biological role of CS and as potential antimicrobials.

## Figures and Tables

**Table 1:** Summary of the derived SAXS data.

Parameter	CysK	CysE	CS
**Guinier Analysis**			
Guinier radius R_g_ (nm)	2.64 ± 0.05	3.90 ± 0.04	6.10 ± 0.11
*I(0)* (cm^−1^)	6.71 ± 0.02	19.08 ± 0.09	27.03 ± 0.23
*q*-range (nm^−1^)	0.22–0.39	0.13–0.31	0.13–0.2
Quality (%)	96	91	99
**GNOM**			
Max. dimension D_max_ (nm)	8.5	13.0	22.0
Guinier radius R_g_ (nm)	2.623 ± 0.006	3.856 ± 0.009	6.606 ± 0.003
*q*-range (nm^−1^)	0.1–3.5	0.1–3.5	0.1–3.5
Porod volume (kDa)	72	189	303
**CRYSOL**			
PDB file	1oas	Hexamer built from 1t3d	
Guinier radius R_g_ (nm)	2.572	3.361	
Molecular weight (kDa)	67.57	171.6	
Max. dimension D_max_ (nm)	8.675	11.0	
**DAMMIF** (def. parameter, 10 runs)			
*q* range for fitting (nm^−1^)	0.12–3.9	0.12–3.9	0.12–3.9
Symmetry, anisotropy assumption	P1, unknown	P1, unknown	P1, unknown
χ^2^ range	1.803–1.833	2.044–2.119	2.077–3.731
Resolution from SASRES (nm)	4.6 ± 0.3	2.9 ± 0.2	9.4 ± 0.2
NSD (standard dev.), no clusters	0.9 (0.12), 1	0.59 (0.01), 1	1.43(0.06), 2
MW estimate for proteins (kDa)	58.6	155	300
Phase radius of gyration (nm)	2.62	3.86	6.61
Maximum phase diameter (nm)	10.500	14.800	24.300
**SASBDB** [83] **codes**	SASDGW6	SASDGV6	SASDGX6

## Supplementary Materials

Supplementary materials can be found at https://www.mdpi.com/1422-0067/20/20/5219/s1. Figure S1: Guinier fits (continuous lines) determined in the q regime given in Table 1, with dots indicating data points of CysK (yellow), CysE (magenta), and CS complex (blue); Figure S2: Dimensionless Kratky plots of the GNOM data shown in Figure 6A. CysK (cyan), CysE (blue) and CS complex (yellow); Figure S3: Ab initio model of the CS complex overlaid with the manual SASpy model. (A) Real space model (B) SAXS data.

## Abbreviations

AcCoA	acetyl coenzyme A
Acid Fuchsin	2-amino-5-[(4-amino-3-sulfophenyl)(4-imino-3-sulfo-2,5-cyclohexadien-1-ylidene)methyl]- 3-methyl-benzenesulfonic acid, sodium salt
ACTH	adrenocorticotropic hormone
ANSA	8-Anilino-1-naphthalenesulfonic acid ammonium salt
AO50	sodium 4-(4-(benzyl-et-amino)-ph-azo)-2,5-di-cl-benzenesulfonate
BSA	bovine serum albumin
DTT	dithiothreitol
EDTA	ethylenediaminetetraacetic acid
Eosin B	4’,5’-dibromo-3’,6’-dihydroxy-2’,7’-dinitro-spiro[isobenzofuran-1(3H),9’-[9H]xanthen]-3one
CS	cysteine synthase
CR	disodium 4-amino-3-[[4-[4-[(1-amino-4-sulfonatonaphthalen-2yl) diazenyl] phenyl] phenyl] diazenyl] naphthalene-1-sulfonate
CysD	ATP sulfurylase
CysE	serine acetyltransferase
CysH	phosphoadenosine phosphosulphate reductase
CysK	*O*-acetylserine sulfhydrylase isozyme A
CysM	*O*-acetylserine sulfhydrylase isozyme B (*O*-phosphoserine sulfhydrylase)
CysN	CysD-associated GTPase
GAPDH	glyceraldehyde 3-phosphate dehydrogenase
HCCA	α-cyano-4-hydroxycinnamic acid
HPLC-SEC	high-pressure liquid chromatography-size exclusion chromatography
IPGT	isopropyl β-D-1-thiogalactopyranoside
MALDI TOF/TOF	matrix-assisted laser desorption ionization-time of flight/time of flight
OAS	*O*-acetylserine
NAS	*N*-acetylserine
PDDF	pair distance distribution function
PLP	pyridoxal 5′-phosphate
PPIs	protein–protein interactions
RBB	disodium 1-amino-9,10-dioxo-4-[3-(2-sulfonatooxyethylsulfonyl)anilino]anthracene-2-sulfonate
SAXS	small angle X-ray scattering
TCEP	tris(2-carboxyethyl)phosphine)
TFA	trifluoroacetic acid
