# Supplementary material for: Combination of SAXS and Protein Painting Discloses the Three-Dimensional Organization of the Bacterial Cysteine Synthase Complex, a Potential Target for Enhancers of Antibiotic Action
Source: Int J Mol Sci. 2019 Oct 21;20(20):5219. doi: 10.3390/ijms20205219 (PMC6829319; doi:10.3390/ijms20205219)
Supplement: Supplementary file 1 [file ijms-20-05219-s001.zip › ijms-574747-supplementary.pdf]

Supplementary Materials

Combination of SAXS and Protein Painting Discloses the Three-Dimensional Organization of the Bacterial Cysteine Synthase Complex, a Potential Target for Enhancers of Antibiotic Action

Brenda Rosa [1], Marialaura Marchetti [2], Gianluca Paredi [3], Heinz Amenitsch [4], Nina Franko [5], Roberto Benoni [1,†], Barbara Giabbai [6], Maria Giovanna De Marino [1,‡], Andrea Mozzarelli [5,7], Luca Ronda [1,7], Paola Storici [6,*], Barbara Campanini [5,8,*] and Stefano Bettati [1,7,8]

[1] Dipartimento di Medicina e Chirurgia, Università di Parma, Via Gramsci 14, 43126, Parma, Italy; brenda.rosa@studenti.unipr.it (B.R.); roberto.benoni@uochb.cas.cz (R.B.); mariagiovanna.demarino@studenti.unipr.it (M.G.D.M.); luca.ronda@unipr.it (L.R.); stefano.bettati@unipr.it (S.B.)
[2] Centro Interdipartimentale Biopharmanet-TEC, Università di Parma, Parco Area delle Scienze 27/A, 43124, Parma, Italy; marialaura.marchetti@unipr.it (M.M.)
[3] Centro Interdipartimentale Siteia, Università di Parma, Parco Area delle Scienze 181/A, 43124, Parma, Italy; gianluca.paredi@unipr.it (G.P.)
[4] Graz University of Technology, Institute for Inorganic Chemistry, Stremayrgasse 9, 8010, Graz, Austria; heinz.amenitsch@elettra.eu (H.A.)
[5] Dipartimento di Scienze degli Alimenti e del Farmaco, Università di Parma, Parco Area delle Scienze 23/A, 43124, Parma, Italy; nina.franko@studenti.unipr.it (N.F.); andrea.mozzarelli@unipr.it (A.M.)
[6] Structural Biology Laboratory, Elettra Sincrotrone Trieste S.C.p.A., SS 14 km 163,5 in AREA Science Park – 34149, Basovizza -Trieste, Italy; barbara.giabbai@elettra.eu (B.G.)
[7] Istituto di Biofisica, CNR, Via Moruzzi 1, 56124, Pisa, Italy
[8] Italian National Institute of Biostructures and Biosystems, Via Medaglie d'Oro 305, 00136, Rome, Italy
* Correspondence: paola.storici@elettra.eu; Tel: +39-0403-758854 (P.S.); barbara.campanini@unipr.it; Tel: +39-0521-906333 (B.C.)
† Current address: Institute of Organic Chemistry and Biochemistry CAS, Flemingovo nam. 2, Praha 6, 16610, Czech Republic
‡ Current address: Dipartimento di Medicina e Chirurgia, Università dell'Insubria, via Guicciardini 9, 21100 Varese, Italy

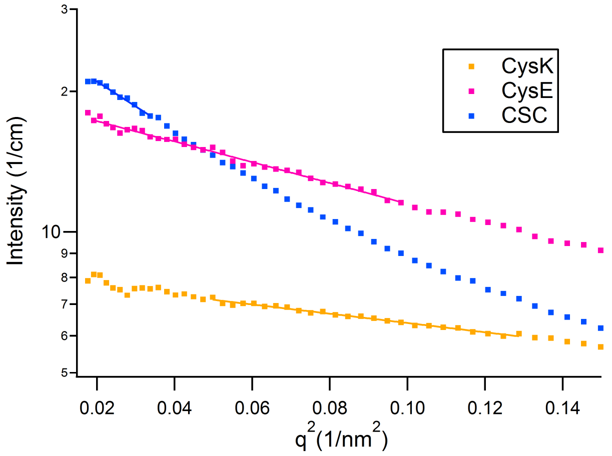

Figure S1. Guinier fits (continuous lines) determined in the q regime given in Table 1 with dots indicating data points of CysK (yellow), CysE (magenta) and CS complex (blue).

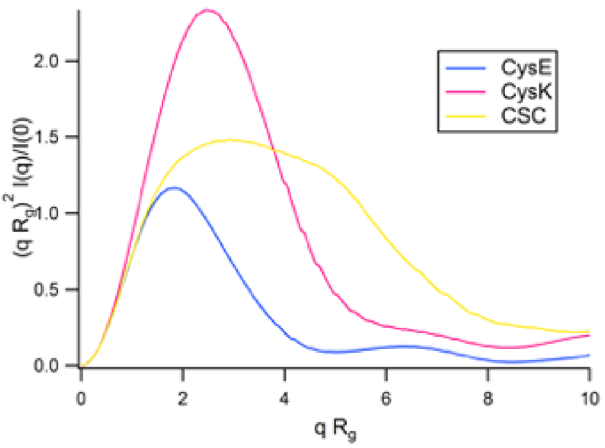

Figure S2. Dimensionless Kratky plots of the GNOM data shown in Figure 6A. CysK (cyan), CysE (blue) and CS complex (yellow).

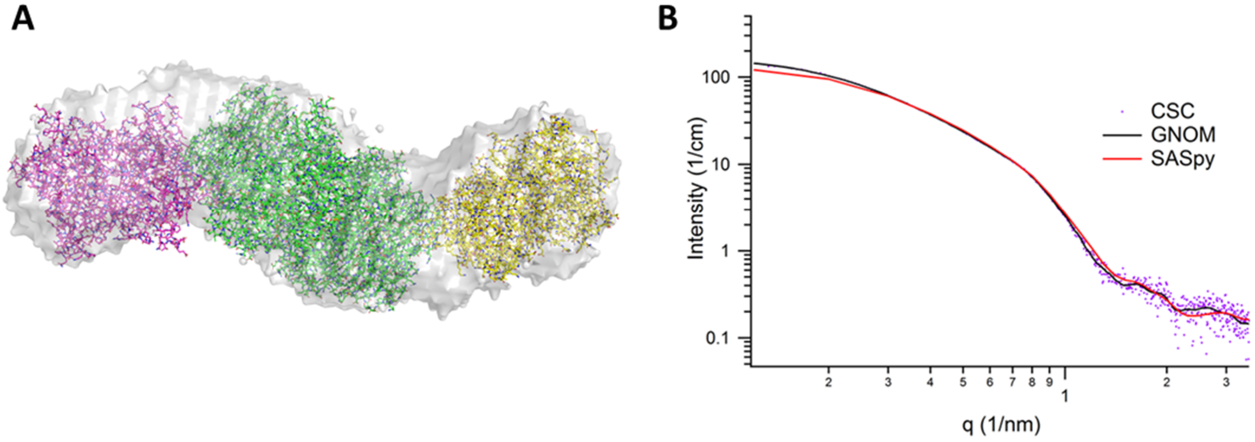

Figure S3. *Ab initio* model of CS complex overlaid with the manual SASpy model. (**A**) Real space model (**B**) SAXS data.
